# Supplementary material for: Automated extraction of chemical synthesis actions from experimental procedures
Source: Nat Commun. 2020 Jul 17;11:3601. doi: 10.1038/s41467-020-17266-6 (PMC7367864; doi:10.1038/s41467-020-17266-6)
Supplement: Supplementary file 1 — Supplementary Information [file 41467_2020_17266_MOESM1_ESM.pdf]

Supplementary Information for "Automated extraction of chemical synthesis actions from experimental procedures"

Alain C. Vaucher et al.

Supplementary Note 1: Properties of actions

The Supplementary Table 1 lists the properties associated with the actions presented in Table 1 of the main article. The variable type "chemical" represents the name of a chemical, to which one or several quantities can be attached. For instance, *"0.036 g of sodium azide (0.56 mmol) was added."* results in an `Add` action, where the property "material" contains the name *"sodium azide"* attached with the quantities *"0.036 g"* and *"0.56 mmol"*.

Supplementary Table 1: Action types and corresponding properties.

Action name	Variable name	Variable type
`InvalidAction`	error	string
`Add`	material	chemical
	dropwise	boolean
	temperature	string (optional)
	atmosphere	string (optional)
	duration	string (optional)
`CollectLayer`	layer	string
`Concentrate`	(none)	
`Degas`	gas	string (optional)
	duration	string (optional)
`DrySolid`	duration	string (optional)
	temperature	string (optional)
	atmosphere	string (optional)
`DrySolution`	material	string (optional)
`Extract`	solvent	chemical
	repetitions	integer
`Filter`	phase_to_keep	string (optional)
`FollowOtherProcedure`	(none)	
`MakeSolution`	materials	list of chemicals
`Microwave`	duration	string (optional)
	temperature	string (optional)
`OtherLanguage`	(none)	
`Partition`	material_1	chemical
	material_2	chemical
`PH`	material	chemical
	ph	string (optional)
	dropwise	boolean
	temperature	string (optional)
`PhaseSeparation`	(none)	
`Purify`	(none)	

`Quench`	material	chemical
	dropwise	boolean
	temperature	string (optional)
`Recrystallize`	solvent	chemical
`Reflux`	duration	string (optional)
	dean_stark	boolean
	atmosphere	string (optional)
`SetTemperature`	temperature	string
`Sonicate`	duration	string (optional)
	temperature	string (optional)
`Stir`	duration	string (optional)
	temperature	string (optional)
	atmosphere	string (optional)
`Triturate`	solvent	chemical
`Wait`	duration	string
	temperature	string (optional)
`Wash`	material	chemical
	repetitions	integer
`Yield`	material	chemical
`NoAction`	(none)	

Supplementary Note 2: Metrics for the finetuning experiments

The Supplementary Figure 1 shows the metrics evaluated on the validation split of the annotation dataset during the finetuning experiments. As is explained in the manuscript (Methods), we selected, from each refinement experiment, the model checkpoint with the highest accuracy on the validation set. The Supplementary Table 2 compares the performance metrics of these models and of a few selected ensemble models.

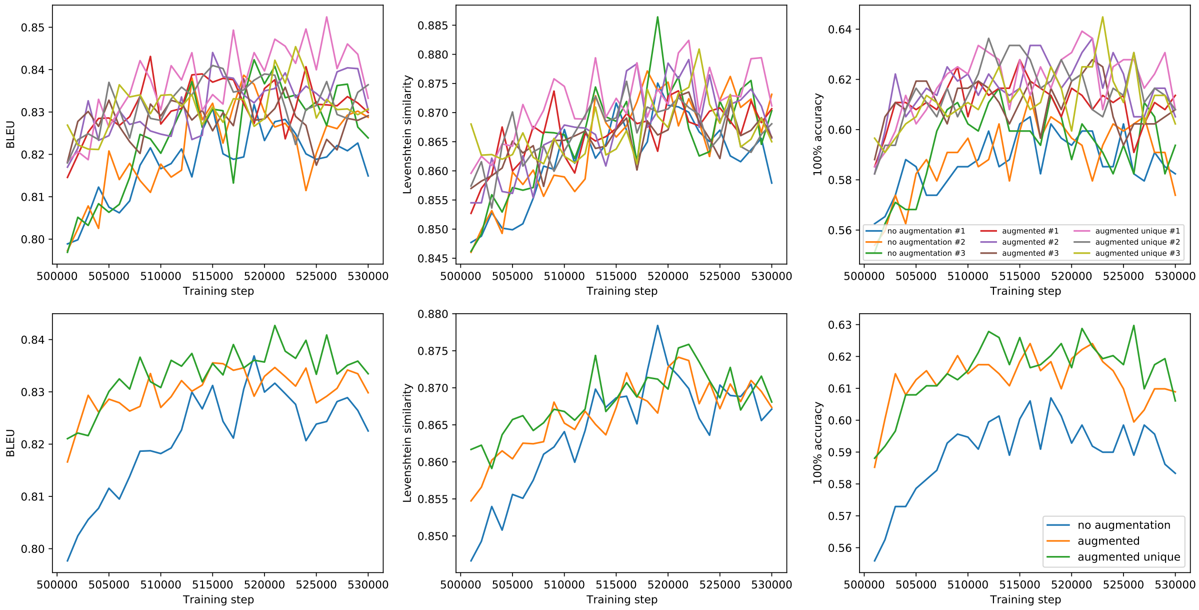

Supplementary Figure 1: Metrics for the machine-learning models during the fine-tuning experiments. Each approach for data augmentation (no augmentation, augmented, augmented unique) was repeated three times with different random number generator seed. For the three plots on top, all the runs are represented by distinct curves. For the three lower plots, the metrics are averaged with respect to the data augmentation approach. For clarity, the values at the beginnig of the finetuning experiment (step 500000) are not shown in the figures. For completeness, we give them here instead: 0.584 (BLEU), 0.710 (Levenshtein similarity), 0.298 (100% accuracy).

The Supplementary Figure 1 and Supplementary Table 2 show that finetuning improves the metrics in all cases. Data augmentation allow the models to reach higher accuracy, especially in terms of BLEU score and accuracy. Removing duplicate samples after data augmentation ("augmented unique") leads to a lower accuracy, but the difference is not significant. The effect of data augmentation is even more pronounced for the models trained

Supplementary Table 2: Performance metrics of all models for the extraction of actions. The metrics are evaluated on the annotation test set. For each data augmentation approach (no augmentation, augmented, augmented unique), three models obtained from different random number generator seeds are listed, as well as an ensemble model combining all three models. All values are given in %, and the best values are indicated in bold.

Model	Validity	BLEU score	Levenshtein similarity	100% accuracy	90% accuracy	75% accuracy
Combined rule-based model	**100.0**	51.5	60.1	21.9	29.0	42.6
Pretrained translation model	**100.0**	58.6	68.7	24.7	33.2	48.3
No pretraining, no augmentation	96.9	22.5	45.9	13.1	15.1	21.9
No pretraining, augmented	98.9	62.6	73.4	34.4	43.2	61.9
No pretraining, augmented unique	98.9	64.7	76.4	37.8	47.7	62.8
Refined, no augmentation, #1 (A)	99.7	81.5	85.7	56.8	67.3	80.4
Refined, no augmentation, #2	99.7	82.7	86.7	56.8	68.8	81.5
Refined, no augmentation, #3	**100.0**	81.5	85.6	55.4	68.2	80.1
Refined, no augmentation, ensemble	99.7	82.7	86.5	58.0	69.3	80.7
Refined, augmented, #1 (B)	99.7	83.7	86.2	60.5	69.9	81.8
Refined, augmented, #2	99.4	84.1	86.4	59.4	70.5	81.8
Refined, augmented, #3	99.4	84.2	86.5	58.0	69.9	82.7
**Refined, augmented, ensemble**	99.4	**85.0**	86.9	**60.8**	**71.3**	82.4
Refined, augmented unique, #1 (C)	99.1	84.3	86.7	59.4	70.5	81.8
Refined, augmented unique, #2	99.1	84.0	86.8	59.7	70.7	82.4
Refined, augmented unique, #3	99.1	84.3	**87.2**	59.1	69.0	**83.5**
Refined, augmented unique, ensemble	99.1	84.2	86.8	59.7	70.2	82.4
Ensemble model of A, B and C	99.7	83.1	86.3	60.2	69.3	81.5

on the annotation dataset only. For three of the four ensemble models, the extraction accuracy is larger than for any of the models in the ensemble. The model selected for analysis and discussion is the model resulting from ensembling the refinement experiments after data augmentation without duplicate removal. The Supplementary Table 2 also shows that models without pretraining do not nearly reach the accuracy of the best refined models.

Supplementary Note 3: Modified formula for the BLEU score

The (unmodified) BLEU score is calculated with the function `corpus_bleu` of the `nltk` Python package (http://www.nltk.org/, `nltk` version 3.4.5). Using this function, all the sentences containing less than four words receive a score of zero. In the context of this work, this would assign a null score to many correct predictions that consist of only one action without parameters, such as `Concentrate`. To avoid this, we pad shorter sentences with an empty token to a length of four. For sentences longer than four words, the unmodified and modified BLEU scores are identical.

The Supplementary Table 3 shows a few examples of unmodified and modified BLEU scores.

Supplementary Table 3: Examples of BLEU score calculation. The `corpus_bleu` column contains the BLEU score as calculated by `nltk`, and the `modified_bleu` column contains the modified BLEU scores as reported in this work.

Ground truth	Prediction	`corpus_bleu`	`modified_bleu`
CONCENTRATE.	CONCENTRATE.	0.0	1.0
CONCENTRATE; PURIFY.	CONCENTRATE; PURIFY.	0.0	1.0
CONCENTRATE; PURIFY.	PURIFY; CONCENTRATE.	0.0	0.0
ADD water; PURIFY.	ADD water; CONCENTRATE; PURIFY.	0.0	0.0
ADD water; WASH with brine; PURIFY.	ADD H2O; WASH with brine; PURIFY.	0.54	0.54

Supplementary Note 4: Unsupported sentences with nonlinear sequences of actions

In our annotation dataset of 1764 sentences, only four sentences correspond to an unsupported nonlinear sequence of actions. They are the following:

- *"Then the reaction mixtures were combined and filtered."*
- *"The solution containing the product obtained previously is then added to this reaction medium very slowly, followed by addition of 23 mL (165 mmol) of triethylamine."*
- *"Separately, under nitrogen gas was in another vessel, a solution was prepared from methyl (S)-2-benzyloxycarbonylamino-3-phenylpropanoate (20.0 g, 63.9 mmol), dibromomethane (22.22 g, 127.8 mmol) and THF (40 mL) (liquor B)."*
- *"The residue and the above solid product were combined and triturated repeatedly with diisopropyl ether."*

In the annotation dataset, an `InvalidAction` is associated to each of these sentences.

Supplementary Note 5: Discussion of the current set of actions

Defining the action types and corresponding properties is a compromise. On the one hand, we want to keep the action vocabulary concise in order to limit the complexity and make the models easy to train. On the other hand, we would like to cover as many operations as possible.

Currently, sentences containing unsupported operations are either converted to an `InvalidAction`, or the incompatible piece of information is ignored. The most common examples are the following:

- Currently, `Add` considers a main reactor containing a reaction mixture to which other substances are added. In some experimental procedures, however, the reaction mixture is poured into another solution instead. While in many cases the directionality of the addition operation does not matter, sometimes this differentiation may be important, such as when the reaction mixture is to be added to something else dropwise. To support this, a property specifying the directionality of the addition could be added to `Add`.

- `SetTemperature` indicates a punctual change in temperature, while in some procedures the change in temperature happens over a specified duration. Such an example would be *"The reaction mixture is cooled to room temperature over 5 hours."* Currently, such a sentence would be converted to a `Stir` action, meaning a punctual change to room temperature followed by stirring for five hours. In most cases, this distinction is irrelevant. Still, to make it explicit, a property for the duration could be added to `SetTemperature`.

- The `Purify` action type currently has no properties. It could be extended to include information about how the purification should proceed.

- Some experimental procedures specify an atmosphere for operations converted to `SetTemperature`.

`SetTemperature` currently has no support for that, but a corresponding property could be added if desired.

- Some operations occurring in experimental procedures are not covered by the current set of actions. This could be remedied by adding corresponding actions types and properties. Examples are hydrogenation, operations related to electrochemistry, or other kinds of irradiation than microwave irradiation.

- For `CollectLayer`, one must specify whether the selected layer is the organic or aqueous one. This limits its scope to aqueous-organic partitioning and does not support organic-organic partitionings such as methanol-hexanes. To improve on this the "layer" property of `CollectLayer` could be extended to also accept solvent names.
