# Supplementary material for: Automated extraction of chemical synthesis actions from experimental procedures
Source: Nat Commun. 2020 Jul 17;11:3601. doi: 10.1038/s41467-020-17266-6 (PMC7367864; doi:10.1038/s41467-020-17266-6)
Supplement: Supplementary file 3 — Description of Additional Supplementary Files [file 41467_2020_17266_MOESM3_ESM.pdf]

Description of Additional Supplementary Files

File Name: Supplementary Data 1
Description: Sentences from the annotation test set with action sequences extracted by the different approaches.

File Name: Supplementary Data 2
Description: Top 5 suggestions predicted by the deep-learning prediction model for sentences from the annotation test set.

File Name: Supplementary Data 3
Description: Detailed guideline for the annotation of experimental procedure sentences.

File Name: Supplementary Data 4
Description: OpenNMT configuration file for pretraining of the translation model.
